# Generation of Novel Human Red Blood Cell-Bearing Humanized Mouse Models Based on C3-Deficient NOG Mice

**DOI:** 10.3389/fimmu.2021.671648

**Published:** 2021-07-27

**Authors:** Takuya Yamaguchi, Ikumi Katano, Iyo Otsuka, Ryoji Ito, Misa Mochizuki, Motohito Goto, Takeshi Takahashi

**Affiliations:** ^1^Laboratory Animal Research Department, Central Institute for Experimental Animals (CIEA), Kawasaki, Japan; ^2^Pathological Analysis Center, CIEA, Kawasaki, Japan; ^3^Animal Resource & Technical Research Center, CIEA, Kawasaki, Japan

**Keywords:** humanized mouse, red blood cell, complements, NOG, macrophage, gadolinium chloride

## Abstract

Despite recent advances in immunodeficient mouse models bearing human red blood cells (hRBCs), the elimination of circulating hRBCs by residual innate immune systems remains a significant challenge. In this study, we evaluated the role of mouse complement C3 in the elimination of circulating hRBCs by developing a novel NOG substrain harboring a truncated version of the murine C3 gene (NOG-C3^ΔMG2-3^). Genetic C3 deletion prolonged the survival of transfused hRBCs in the circulation. Chemical depletion and functional impairment of mouse macrophages, using clodronate liposomes (Clo-lip) or gadolinium chloride (GdCl_3_), respectively, further extended the survival of hRBCs in NOG-C3^ΔMG2-3^ mice. Low GdCl_3_ toxicity allowed the establishment of hRBC-bearing mice, in which hRBCs survived for more than 4 weeks with transfusion once a week. In addition, erythropoiesis of human hematopoietic stem cells (hHSCs) was possible in NOG-C3^ΔMG2-3^/human GM-CSF-IL-3 transgenic mice with Clo-lip treatment. These findings indicate that mouse models harboring hRBCs can be achieved using NOG-C3^ΔMG2-3^ mice, which could facilitate studies of human diseases associated with RBCs.

## Introduction

Xenotransplantation in immunodeficient mice has become an essential *in vivo* model for studying human hematopoietic cell development and hematological diseases (1, 2). The most commonly used immunocompromised mouse strains are NOD/Shi-scid/IL-2Rγ^null^ (NOG) (3), NOD/LtSz-scid/IL-2Rγ^null^ (NSG) (4), and BALB/c-Rag2^null^/IL-2Rγ^null^ (5). In the last two decades, significant efforts have been made to expand the repertoire of cell lineages (6–10) and engraft human cells to establish a functional humanized immune system (11–14). As a result, numerous mouse strains recapitulating human diseases are now available for drug discovery (15–18). However, some human hematopoietic lineages cannot be generated using the current humanized mouse models.

Human red blood cells (hRBCs) are the most abundant cells in the blood, comprising approximately 40% of the hematocrit. However, hRBCs are short-lived in experimental models, hindering progress in malaria research (19–21). The rate of differentiation of hematopoietic stem cells (HSCs) to mature hRBCs is extremely low in humanized mouse models, even though immature human erythrocytes can be found in the bone marrow (BM) of human hematopoietic stem cell (hHSC)-reconstituted NOG or NSG mice (Dr. Tatsutoshi Nakahata, personal communication) (22). The absence of mature hRBCs in the peripheral blood (PB) was also reported in the novel NSG-W41 model, which has an enhanced ability for human hematopoiesis (7, 22). Although the success of induction of hRBCs was reported in a dual humanized mouse model with human liver and hematopoietic systems during the submission of this manuscript, the frequency in total RBCs remained not more than 5% (23).

In addition, when immunodeficient mice are transfused with large amounts of mature hRBCs (i.e., when 20–40% of erythrocytes in the blood circulation are human-derived), the erythrocytes are rapidly eliminated within a few days (24, 25). Repeated injections of hRBCs every 1–2 days are necessary to overcome the poor hRBC retention and establish hRBC-bearing mice (20, 26–28). Hu et al. aimed to produce an improved hRBC-bearing mouse model by depleting macrophages and suppressing macrophage-mediated hRBCs phagocytosis. Selective depletion of macrophages using clodronate liposomes (Clo-lip) not only prolonged the survival of injected hRBCs but also allowed the development of circulating hRBCs from hHSCs in the blood of NOD/SCID and NSG mice (24). Although this model can be used for hRBC studies, repeated injections of Clo-lip are toxic or even lethal. In addition, the frequent replenishment of hRBCs by repeated daily transfusion may not recapitulate the physiology of hRBCs, indicating that current mouse models are not always suitable for long-term studies, such as modeling the life cycle of malaria parasites.

The fact that murine macrophages rapidly eliminate transfused mature hRBCs suggests that hRBCs are recognized by host innate immune cells as foreign substances by either direct or indirect mechanisms. Glycans, such as oligomannoside- and N-acetyllactosamine-type glycans, have been suggested to play a role in the recognition of hRBCs by mouse innate immune cells (29). Ishihara et al. demonstrated that mouse C3 can deposit on hRBCs and suggested the role of C3 in the elimination of hRBCs (30). In addition, Chen et al. reported that, in hRBC-transplanted NOD/SCID mice, the complement induces hRBC adherence to murine phagocytes (25), suggesting that hRBCs are opsonized by the murine complement, leading to hRBC recognition by murine macrophages.

In this study, we investigated the role of murine complement C3 in the recognition and rejection of hRBCs *in vivo* by establishing a novel NOG substrain harboring a truncated version of the murine C3 gene (NOG-C3^ΔMG2-3^). The lack of C3 prolonged the survival of exogenous hRBCs in the circulation. Gadolinium chloride (GdCl_3_) was reported to inhibit macrophages in the rat (31, 32) and mouse liver (33). To develop an improved method for depleting or suppressing mouse macrophages, we compared the ability of GdCl_3_ and Clo-lip to inhibit macrophages in NOG-C3^ΔMG2-3^ and NOG mice. GdCl_3_ and Clo-lip significantly prolonged the survival of transfused hRBCs in NOG-C3^ΔMG2-3^ mice. Although the effects of GdCl_3_ were weak compared with Clo-lip, GdCl_3_ was less toxic than Clo-lip; thus, transfusion of hRBCs with GdCl_3_ treatments once a week maintained hRBCs for an extended period. Finally, induction of hRBCs from hHSCs was achieved in hHSC-reconstituted NOG-C3^ΔMG2-3^/human (h) GM-CSF-IL-3 (GM3) transgenic (Tg) mice after Clo-lip treatment.

The results indicate that NOG-C3^ΔMG2-3^ mice practically facilitate the production of humanized mice with hRBCs and they will be a novel model to study malaria infection and other human erythrocyte-related diseases.

## Materials and Methods

### Mice

NOG (NOD.Cg-Prkdc^scid^ Il2rg^tm1Sug^/ShiJic) (3) and NOG-C3^ΔMG2-3^ (NOD.Cg-Prkdc^scid^ Il2rg^tm1Sug^C3^em1^/Jic) mice were maintained at the Central Institute for Experimental Animals (CIEA) under specific pathogen-free conditions. To generate NOG-C3^ΔMG2-3^ mice, we used the CRISPR/Cas9 system for genome editing (34, 35). Four different guide RNA (gRNA) sequences were designed to target exons 5, 6, and 7 of the complement 3 gene. All gRNAs were cloned into the PX330 plasmid (36) and cleavage activity was confirmed using the reporter construct pCAGGS-EGxxFP (37). Both plasmids were obtained from Addgene (Watertown, MA). The genomic region containing the target exons was inserted in the middle of the EGFP gene, and the plasmid was transfected into HEK293T cells together with each gRNA-PX330 plasmid. Genome-editing success was evaluated *via* GFP signal rescue after cleavage of the targeted exon. Two gRNAs (gRNA1 and -4) were selected, and a mixture of the two was used for microinjection into fertilized eggs of NOG mice. The sequences of the gRNAs were as follows: gRNA1 targeting exon 5, 5’-CTTGACAGGAATGCCATCGG-3’gRNA4 targeting exon 7, 5’-CATCGATGACCCAAATGGCC-3’.

### Ethics Statement

All studies involving human participants were reviewed and approved by the research ethics committee of the CIEA. Study participants provided written informed consent. All animal experiments were performed in accordance with institutional guidelines (14038, 17024, and 20043) and were approved by the animal experimentation committee of the CIEA.

### Flow Cytometry and Antibodies

Murine PB was collected from the retro-orbital venous plexus using heparinized pipettes periodically under anesthesia. BM cells were obtained by flushing femurs with 1 mL phosphate-buffered saline (PBS). PB and BM samples were diluted 10- and 5-fold with PBS, respectively, and 10 μL of the diluted sample was mixed with an equal volume of antibody mixture (described below). After 20–30 min of incubation in the dark at room temperature, the samples were further diluted 15–20-fold with PBS and analyzed on a FACSCanto™ or LSRFortessa™ flow cytometer (BD Biosciences, San Jose, CA). Flow cytometry data were analyzed using FlowJo (ver. 10.7.1, BD Biosciences).

Anti-human CD235a (glycophorin A)-allophycocyanin/cyanine 7 (APC/Cy7), anti-human CD71-APC and anti-mouse TER-119-phycoerythrin (PE) were purchased from BioLegend (San Diego, CA). Fluorescent isothiocyanate (FITC)-labeled goat anti-mouse complement C3 polyclonal antibody was purchased from MP Biomedicals (Santa Ana, CA). Anti-mouse complement component C1q-PE and anti-mouse complement component C4-biotin antibodies were purchased from Cedarlane (Burlington, ON). Streptavidin-PE (StAv-PE) was purchased from BD Bioscience.

### Enzyme-Linked Immunosorbent Assay (ELISA)

C3 levels in the plasma were measured using an anti-mouse complement C3 ELISA kit (Abcam, Cambridge, UK) according to the manufacturer’s instructions.

### Transplantation of hRBCs

We collected PB from healthy donors to obtain hRBCs for transfusion. The plasma and buffy coat were removed after centrifugation (400 × g for 5 min). The pellets were washed with PBS, and the RBCs were adjusted to a concentration of 1 × 10^10^ cells/mL. Mice were intravenously (i.v.) injected with hRBCs *via* the tail vein. For long-term experiments, the hRBCs were intraperitoneally (i.p.) injected once a week for maintenance after the initial transfusion.

### Transplantation of hHSCs

To reconstitute the human hematopoietic system, 6–8-week-old NOG-C3^ΔMG2-3^, NOG/hGM3 Tg, and NOG-C3^ΔMG2-3^/hGM3 Tg mice were irradiated with X-rays at 160 cGy (MBR-1520R-4; Hitachi, Hitachi, Japan). Then, 5.0 x 10^4^ or 2.5 × 10^4^ umbilical cord blood-derived CD34^+^ cells (StemExpress, Folsom, CA), for NOG-C3^ΔMG2-3^ mice or for NOG/hGM3 Tg and NOG-C3^ΔMG2-3^/hGM3 Tg mice, respectively, were transplanted by i.v. injection the next day; hHSC-NOG-C3^ΔMG2-3^, hHSC-NOG/hGM3 Tg, and hHSC-NOG-C3^ΔMG2-3^/hGM3 Tg mice were thus obtained.

### Chemical Treatment

Clo-lip (400 μL/kg; Hygieia Bioscience, Osaka, Japan) and GdCl_3_ (30 mg/kg; G7532, Sigma Aldrich, St. Louis, MO) were i.v.-injected into 6–10-week-old NOG or NOG-C3^ΔMG2-3^ mice *via* the tail vein. The injection volume was adjusted to 200 μL with saline. Control mice were injected with 200 μL of saline. Mice were injected with Clo-lip, GdCl_3_, or saline four times at 3–4-day intervals and were transfused with hRBCs on the day after the last injection. For long-term experiments, Clo-lip was i.v.-injected as the first treatment to ensure macrophage depletion. Thereafter, GdCl_3_ was administered three times by i.v. injection, at 3–4-day intervals. After the first transfusion of hRBCs, GdCl_3_ was administered by i.p. injection every 4–6 days for maintenance. For the induction of hRBC from hHSCs, hHSC-transplanted mice were treated with Clo-lip as described above with 3-4-days intervals at 8 weeks after hHSC-transplantation.

### *In Vitro* C3 Deposition Assay

The mouse PB (5 μL) was diluted with 50 μL of PBS containing heparin; 3 μL of the solution was mixed with 30 μL of mouse serum from NOG or NOG-C3^ΔMG2-3^ mice and incubated for 30 min at 37°C. Subsequently, samples were stained with FITC-labeled goat anti-C3 polyclonal antibody for 20 min at room temperature. Then, the cells were washed with PBS and analyzed using a FACSCanto flow cytometer.

### *In Vivo* C3 Deposition Assay

Mice were administered hRBCs (2–5 × 10^9^) by i.v. injection and the PB was collected 3 h after the injection. The blood (10 μL) was immediately transferred to a tube containing 100 μL of cold PBS with 5 IU/mL heparin and 10 mM EDTA (PBS/heparin/EDTA) to prevent complement activation. The diluted blood (10 μL) was stained with APC/Cy7-anti-human CD235a and FITC-anti-mouse C3 polyclonal antibodies, or APC/Cy7-CD235a and biotin-anti-mouse C1q or -C4 antibodies, followed by StAv-PE for 20 min on ice. After staining, the cells were suspended in 100 μL of cold PBS/EDTA and analyzed using flow cytometry.

### Histology

Tissues from mice were fixed in 10% neutralized formalin (Mildform, FUJIFILM Wako Pure Chemical, Osaka, Japan). Formalin-fixed tissues were embedded in paraffin and analyzed with either hematoxylin and eosin staining or immunohistochemistry using anti-mouse CD68 antibody (Cell Signaling Technology, Danvers, MA) or anti-F4/80 antibody (BIO-RAD, Hercules, CA). Sections were stained using a fully automated BOND-MAX system (Leica Biosystems, Mount Waverley, Australia). The images were captured using a NanoZoomer S60 scanner (Hamamatsu Photonics, Hamamatsu, Japan).

### Statistical Analysis

The statistical significance of the results was determined using two-way repeated-measures ANOVA or Mixed-effects analysis using GraphPad Prism software (ver. 9.0; GraphPad Software Inc., San Diego, CA).

## Results

### Deposition of Mouse Complement on hRBC Surfaces in NOG Mice

**Figure 1:**
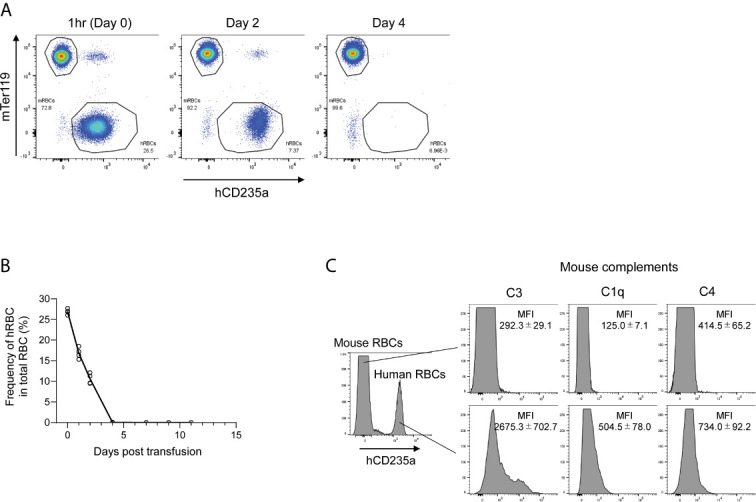
The fate of hRBCs in NOG mice. **(A)** PB was collected from NOG mice at 1 h, 2 days, and 4 days after hRBC transfusion. Human and mouse RBCs were distinguished based on hCD235a (glycophorin A) and Ter119 expression. A representative FACS plot is shown. **(B)** Kinetics of transfused hRBCs in NOG mice. PB was collected at the indicated time points, and the presence of hRBCs was assessed using flow cytometry. The value on day 0 represents the frequency of hRBCs relative to total RBCs at 1 h after transfusion. Each dot represents a value from each mouse, and the line indicates the time course of the means. **(C)** Deposition of murine C3 on the surfaces of transfused hRBCs. PB was collected from hRBC-transfused NOG mice at 3 h post-transfusion, stained with anti-human CD235a and anti-mouse C3, C1q, or C4 antibodies, and analyzed using flow cytometry. The upper histogram represents mouse (hCD235a^-^) RBCs; human (hCD235a^+^) RBCs are shown in the lower histogram.

To examine the fate of transfused hRBCs in NOG mice, we i.v.-injected fresh hRBCs into NOG mice and investigated their dynamics in the circulation using flow cytometry. We found that hRBCs were rapidly eliminated from the circulation of NOG mice, even when 5.0 × 10^9^ hRBCs (equivalent to 25%–35% of total RBCs) were administered (**Figures 1A, B**). Mouse innate immune cells, especially macrophages, have been implicated in the rapid clearance of hRBCs (24); hence, we investigated the role of mouse complement molecules in the elimination of hRBCs by mouse innate immune cells. To evaluate the deposition of mouse complement molecules on the hRBC surfaces, PB was collected 3 h after hRBC injection and stained with polyclonal anti-mouse C1q, C3, or C4b antibodies. Significant amounts of mouse C3 fragments were detected on the transfused hRBC surfaces, whereas C1q and C4 were modest (**Figure 1C**).

### Generation of C3-Deficient NOG Mice

The deposition of mouse C3 on the surfaces of hRBCs indicates that murine C3 may function as an opsonin to facilitate the recognition of hRBCs by mouse innate immune cells. To evaluate the relevance of C3 to hRBC recognition, we disrupted the mouse C3 gene using CRISPR/Cas9. Among five founder mice, one mouse had a 252-nucleotide (nt) deletion resulting in the total exclusion of exon 6 and exclusion of large parts of exons 5 and 7 (nt 614–865; **Supplementary Figure 1**). The resulting 84-amino-acid (aa) truncation (aa 172–255 in pro C3 molecules) corresponded to a part spanning the MG2-MG3 domains in the C3α chain (38). Despite the in-frame deletion, mouse C3 protein was not detected in the plasma by ELISA (**Supplementary Figure 2A**), indicating the absence of mature functional mouse C3 molecules in the circulation. Indeed, when the serum from NOG or mutant (NOG-C3^ΔMG2-3^) mice was incubated with hRBCs *in vitro*, large amounts of mouse C3 or its derivative fragments were deposited on the hRBC surfaces with the NOG mouse serum. By contrast, no signal was detected when hRBCs were incubated with NOG-C3^ΔMG2-3^ mouse serum (**Supplementary Figure 2B**), confirming that NOG-C3^ΔMG2-3^ are C3-deficient.

### Prolonged Survival of hRBCs in NOG-C3^ΔMG2-3^ Mice

**Figure 2:**
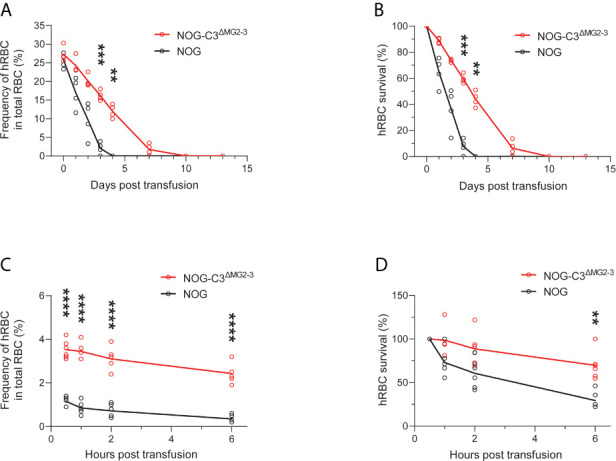
Elimination of hRBCs in NOG and NOG-C3^ΔMG2-3^ mice. **(A, C)** Changes in the frequencies of hRBCs relative to total RBCs over time. PB samples from NOG and NOG-C3^ΔMG2-3^ mice were collected and analyzed at the indicated time points (same as in **Figure 1**). Each group (red line for NOG-C3^ΔMG2-3^ and black line for NOG) consisted of four mice **(A)** or six mice **(C)**. The numbers of transfused hRBCs were 5.0 x 10^9^ **(A)** and 2.5 x 10^8^ **(C)**. **(B, D)** Survival rates of hRBCs. The values in **(B, D)** were calculated from **(A, C)**, respectively, as percentages relative to the value on day 0. Asterisks indicate statistically significant differences between NOG-C3^ΔMG2-3^ and NOG mice as determined by two-way repeated-measures ANOVA (****p < 0.0001, ***p < 0.001 and **p < 0.01).

We then assessed the survival of transfused hRBCs in NOG and NOG-C3^ΔMG2-3^ mice. To this end, we transfused 5.0 × 10^9^ hRBCs into NOG and NOG-C3^ΔMG2-3^ mice and evaluated their dynamics in the circulation using flow cytometry. In NOG mice, the frequency of circulating hRBCs decreased dramatically within a few days (**Figures 1** and **2**). The survival rates of hRBCs were 64.8% ± 11.2% at 1 day post-transfusion (dpt), 36.5% ± 16.3% at 2 dpt, and 7.7% ± 5.9% at 3 dpt; no hRBCs were detected after 4 dpt (**Figure 2B**). The elimination of transfused hRBCs was slower in NOG-C3^ΔMG2-3^ mice than in NOG mice, with hRBC survival rates of 88.8% ± 2.0% at 1 dpt, 73.7% ± 1.2% at 2 dpt, and 59.5% ± 3.4% at 3 dpt (**Figure 2B**). Remarkably, 44.0% ± 5.8% of the transfused hRBCs (equivalent to ~12% of the total RBCs in the mouse blood) were maintained at 4 dpt in NOG-C3^ΔMG2-3^ mice (**Figure 2A**); a significant amount of hRBCs could still be detected up to 7 days after a single transfusion (**Figure 2B**). The transfusion of a smaller number of hRBCs (2.5 × 10^8^) was also tested. The frequency of hRBCs 30 min after transfusion was 1.15% ± 0.21% in NOG mice and 3.53% ± 0.42% in NOG-C3^ΔMG2-3^ mice, which was statistically significant. At 6 hours post transfusion, there were a few hRBCs in NOG mice, whereas 2.43% ± 0.44% of the total RBCs were hRBCs in NOG-C3^ΔMG2-3^ mice (**Figure 2C**). The decrease of the survival rate of hRBCs was more rapid in NOG mice than in NOG-C3^ΔMG2-3^ mice (**Figure 2D**), suggesting that C3-dependent elimination mechanisms are independent of the number of hRBCs. The prolonged survival of human RBCs in NOG-C3^ΔMG2-3^ mice was observed irrespective of the donor blood type because we obtained similar results with different donor blood types (A-type, O-type and AB-type, data not shown).

### Depletion of Mouse Macrophages by Clo-lip Extends the Survival of hRBCs in NOG and NOG-C3^ΔMG2-3^ Mice

**Figure 3:**
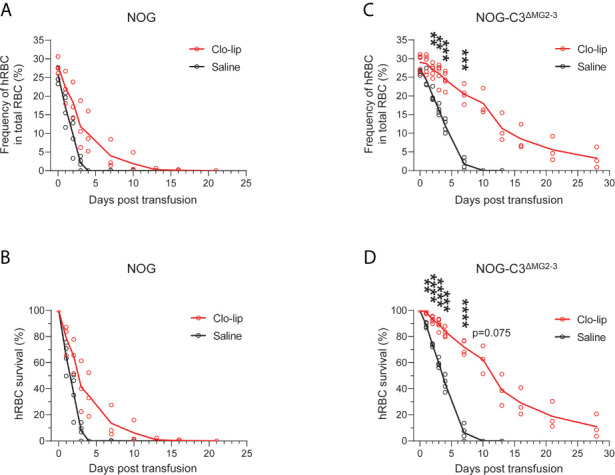
Prolonged survival of hRBCs after Clo-lip treatment. NOG and NOG-C3^ΔMG2-3^ mice were treated with Clo-lip four times at 3–4-day intervals. Mice were transfused with hRBCs at 1 day after the last Clo-lip treatment. Frequencies **(A, C)** and survival rates **(B, D)** of transfused hRBCs in NOG mice **(A, B)** and NOG-C3^ΔMG2-3^ mice **(C, D)**. The NOG group consisted of four saline-treated and four Clo-lip-treated mice. The NOG-C3^ΔMG2-3^ group consisted of four saline-treated and five Clo-lip-treated mice. Two Clo-lip-injected NOG mice (on days 0 and 28) and two Clo-lip-injected NOG-C3^ΔMG2-3^ mice (on days 7 and 10) died during the experiment. Asterisks indicate statistically significant differences between Clo-lip-injected mice and saline-injected mice as determined using Mixed-effects analysis (****p < 0.0001, ***p < 0.001, and **p < 0.01).

Despite the extended hRBC survival in NOG-C3^ΔMG2-3^ mice, the survival period of erythrocytes was significantly shorter than their natural lifespan. Hence, we depleted mouse macrophages by treating NOG and NOG-C3^ΔMG2-3^ mice with Clo-lip, which has been previously shown to extend hRBC survival. After four Clo-lip injections at 3–4-day intervals, we transfused hRBCs into NOG and NOG-C3^ΔMG2-3^ mice. Consistent with the previous reports, Clo-lip extended the survival of hRBCs in NOG mice. We detected 34.7% ± 16.8% and 13.5% ± 12.1% of the initial amount of hRBCs at 4 and 7 dpt, respectively (**Figures 3A, B**). The half-life of hRBCs extended from 1–2 days to 2–3 days in NOG mice; nevertheless, the difference between Clo-lip-treated NOG mice and saline-treated control mice was not statistically significant (**Figure 3B**). By contrast, the extension of hRBC survival was profound in NOG-C3^ΔMG2-3^ mice. The survival rates of hRBCs were 71.9% ± 5.6% at 7 dpt, 38.9% ± 12.0% at 13 dpt, and 18.9% ± 10.1% at 21 dpt (**Figure 3C**); hRBC half-life was extended from 3–4 days to 10–13 days (**Figure 3D**). Notably, a significant number of hRBCs could be detected up to 1 month after a single transfusion of hRBCs.

### GdCl_3_ Prolongs hRBC Survival Without Causing Phenotypic Abnormalities

Despite the prolonged hRBC survival in NOG and NOG-C3^ΔMG2-3^ mice after Clo-lip treatment, Clo-lip caused significant toxicity. Two of four NOG mice and two of five NOG-C3^ΔMG2-3^ mice died during the experiments (**Figure 3**), and the remaining mice exhibited aberrant phenotypes, including ruffled hair, weight loss, and a hunched posture (data not shown). Hence, alternative chemicals are required to either deplete or suppress mouse macrophages.

**Figure 4:**
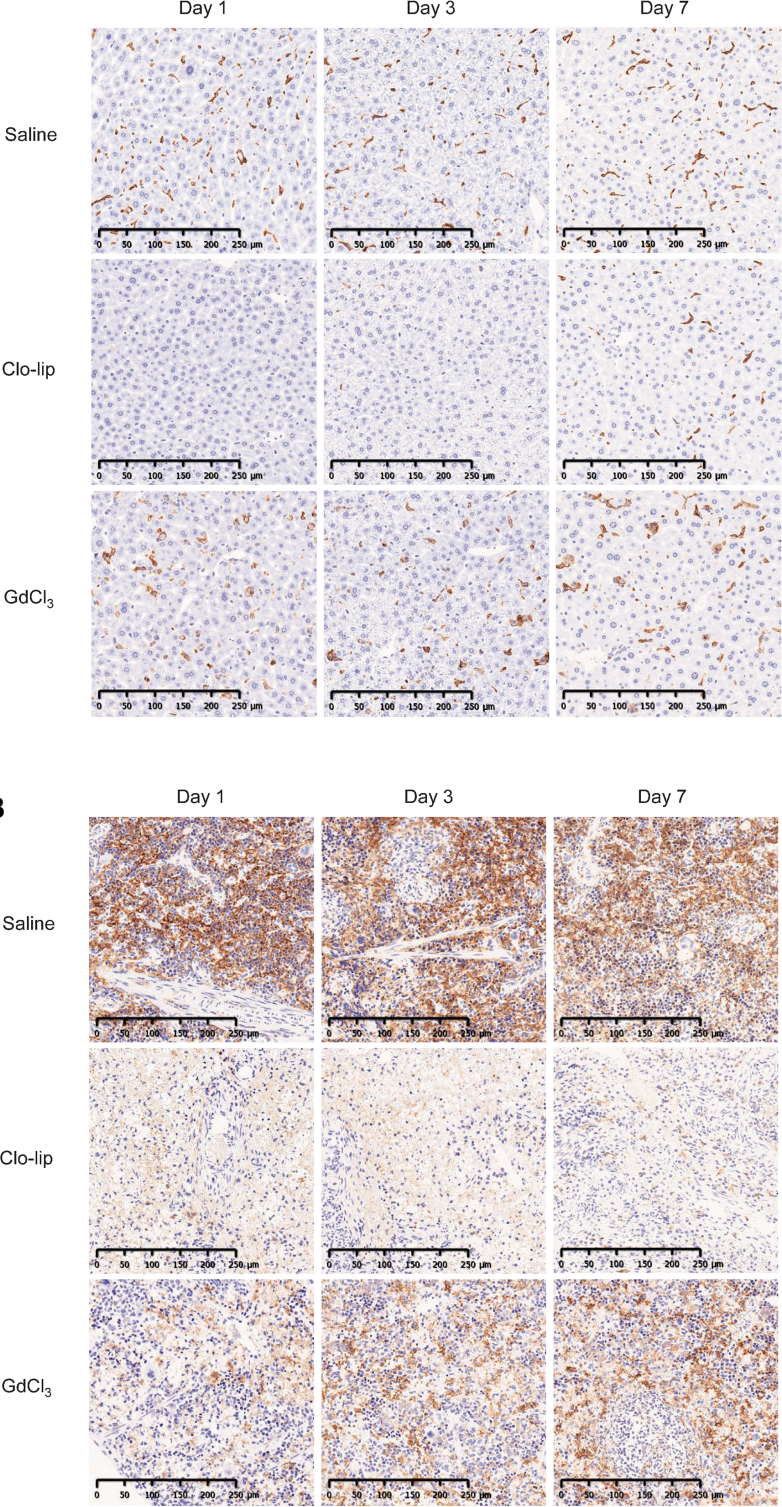
Effects of Clo-lip and GdCl_3_ on macrophages in NOG mice. NOG mice were administered saline, Clo-lip, or GdCl_3_ four times at 3–4-day intervals. Livers **(A)** and spleens **(B)** were excised at 1, 3, or 7 days after the last treatment. Tissue macrophages were detected with anti-F4/80 antibody and visualized using diaminobenzidine (brown). Representative images are shown.

GdCl_3_ has been shown to deplete or suppress macrophages in rats (31) and mice (33). In this study, we administered 30 mg/kg of GdCl_3_ four times at 3–4-day intervals. This dosing was determined based on preliminary results from titration experiments (data not shown). Immunohistochemistry showed that Clo-lip induced severe depletion of F4/80-positive macrophages in the spleen and liver of NOG mice (**Figure 4**). Recovery of hepatic macrophages was detected at 7 days post Clo-lip treatment, and a few macrophages were detected in the spleen. In contrast to Clo-lip, GdCl_3_ did not deplete macrophages in the liver of NOG mice, although there were some morphological changes such as swelling (**Figure 4A**). GdCl_3_ also induced a mild reduction of macrophages in the spleen (**Figure 4B**). Notably, GdCl_3_ treatment did not cause significant toxicity, in sharp contrast to Clo-lip. GdCl_3_-treated mice did not develop overt phenotypic abnormalities, and, although the mice experienced a slight weight loss early after GdCl_3_ treatment, mouse weight returned to physiological levels at 1 week after treatment (**Supplementary Figure 3**).

**Figure 5:**
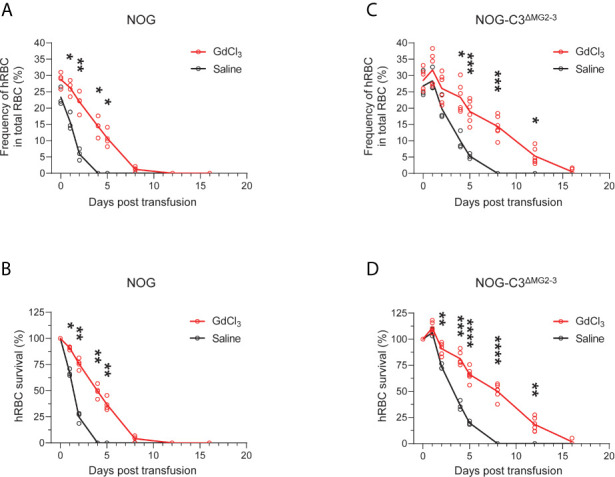
Prolonged survival of hRBCs in NOG-C3^ΔMG2-3^ mice treated with GdCl_3_. NOG and NOG-C3^ΔMG2-3^ mice were treated with GdCl_3_ four times at 3–4-day intervals. Mice were then transfused with hRBCs at 1 day after the last Clo-lip treatment. Frequencies **(A, C)** and survival rates **(B, D)** of transfused hRBCs in NOG mice **(A, B)** and NOG-C3^ΔMG2-3^ mice **(C, D)**. The NOG group consisted of three saline-treated and four Clo-lip-treated mice. The NOG-C3^ΔMG2-3^ group consisted of three saline-treated and six Clo-lip-treated mice. Asterisks indicate statistically significant differences between Clo-lip-injected mice and saline-injected mice as determined using two-way repeated-measures ANOVA (****p < 0.0001, ***p < 0.001, **p < 0.01, and *p < 0.05).

Importantly, GdCl_3_ treatment of NOG and NOG-C3^ΔMG2-3^ mice before hRBC transfusion significantly extended the survival of hRBCs. In NOG mice, hRBC half-life was extended from 1–2 days to approximately 4 days after transfusion (**Figures 5A, B**). The extension of hRBC survival was confirmed in NOG-C3^ΔMG2-3^ mice, where GdCl_3_ increased hRBC half-life from 2–4 days to nearly 8 days. At 12 dpt, 18.3% ± 6.6% of the initial hRBC count was retained, which was equivalent to ~5% of the total RBC count in the mouse blood (**Figures 5C, D**). The effects of GdCl_3_ were also confirmed in NOG mice with a smaller number of hRBCs (2.5 × 10^8^) (**Supplementary Figure 4**).

Notably, GdCl_3_ did not cause significant toxicity in NOG or NOG-C3^ΔMG2-3^ mice, and no deaths occurred during the experiments (data not shown).

### Long-Term Maintenance of hRBCs by Multiple Injections in NOG-C3^ΔMG2-3^ Mice

**Figure 6:**
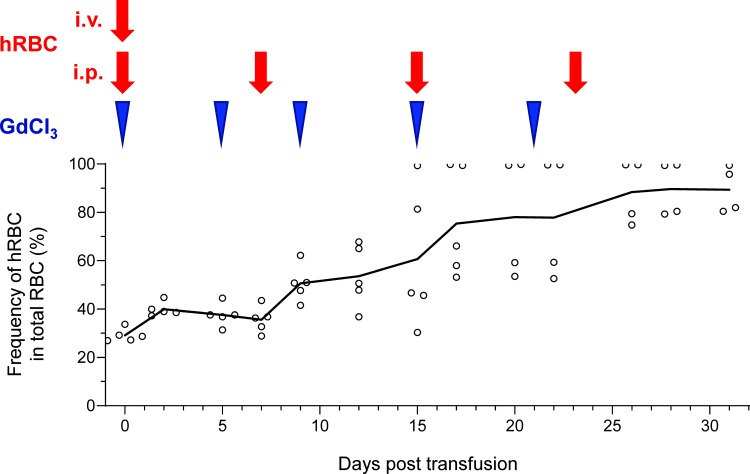
Long-term stable maintenance of hRBCs by repeated administration of hRBCs and GdCl_3_ in NOG-C3^ΔMG2-3^ mice. NOG-C3^ΔMG2-3^ mice were treated with Clo-lip once, and subsequently with GdCl_3_ three times at 3–4-day intervals. Mice were transfused with i.v. (5.0 × 10^9^) and i.p. (5.0 × 10^9^) injections of hRBCs on the next day of the pretreatment cycle, to increase the initial number of hRBCs and avoid transient polycythemia. GdCl_3_ was administered every 4–6 days to suppress murine macrophages. hRBCs were i.p.- injected every 7–8 days. The NOG-C3^ΔMG2-3^ group consisted of five mice. The average numbers of hRBCs at the indicated time points are shown, along with the values from each individual mouse. Blue arrow heads and red arrow represent administration of GdCl_3_ and hRBC supplementation, respectively.

After confirming prolonged survival of hRBCs in NOG-C3^ΔMG2-3^ mice, multiple injections of hRBCs and GdCl_3_ were administered to maintain the level of hRBCs in the circulation for an extended period. To ensure initial elimination of mouse macrophages, Clo-lip was administered as the initial treatment, followed by three GdCl_3_ treatments at 3–4-day intervals. After pretreatment, 5.0 × 10^9^ hRBCs were administered to NOG mice on day 0, and GdCl_3_ was administered on days 2, 5, and 8 by i.p. injection. Consecutive administration of GdCl_3_ did not prolong the survival of hRBCs in NOG mice. The additional administration of 5.0 × 10^9^ hRBCs by i.p. injection on day 5 transiently increased the amount of hRBCs on the following day; however, they rapidly decreased thereafter (**Supplementary Figure 5**). NOG-C3^ΔMG2-3^ mice showed better results. After pretreatment, 5.0 × 10^9^ hRBCs were administered both by i.v. and i.p. injections on day 0, to increase the initial loading amount. Thereafter, GdCl_3_ was i.p.-injected every 4–6 days, and 5.0 × 10^9^ hRBCs were administered by i.p. injection once a week for 3 weeks. The proportion of hRBCs in PB was around 25% after the first injection of hRBCs, and gradually increased with every hRBC injection (**Figure 6**). The proportion of hRBCs in two mice reached nearly 100% by day 15 and was maintained for 2 weeks. The other two mice showed about 80% chimerism by day 28. During the experiment, one mouse died on day 20; all of the other mice were healthy. These results indicate that NOG-C3^ΔMG2-3^ mice are significantly superior with respect to the maintenance of hRBCs compared with NOG mice.

### Induction of Human RBCs From Human HSCs

**Figure 7:**
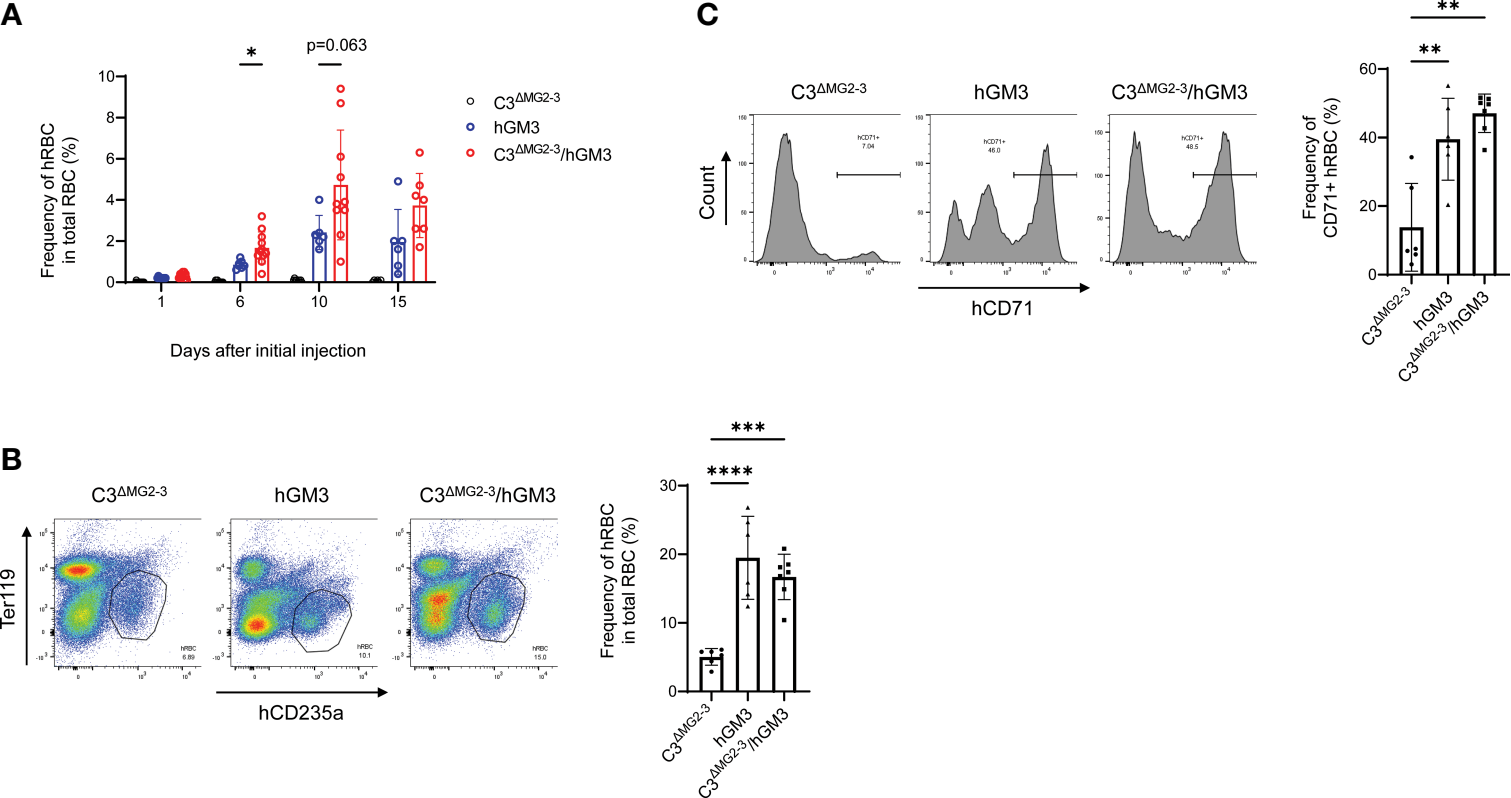
Induction of hRBCs from hHSCs in NOG-C3^ΔMG2-3^/hGM3 Tg mice. **(A)** Development of hRBCs in hHSC engrafted mice. hHSC-NOG-C3^ΔMG2-3^/hGM3 Tg (n=13), hHSC-NOG-C3^ΔMG2-3^ (n=15), or hHSC-NOG/hGM3 Tg (n=10) mice were treated with Clo-lip 8 weeks after HSC transplantation. PB was collected at the indicated time points and analyzed using flow cytometry. Note that six, nine and four in the hHSC-NOG-C3^ΔMG2-3^/hGM3 Tg, hHSC-NOG-C3^ΔMG2-3^, or hHSC-NOG/hGM3 Tg died during the experiments. Asterisks indicate statistically significant differences between hHSC-NOG-C3^ΔMG2-3^ and hHSC-NOG/hGM3 Tg mice as determined using Mixed-effects analysis (****p < 0.0001, **p < 0.01, and *p < 0.05). **(B, C)** Analysis of BM. BM cells from mice in **(A)** were collected 15 days after the initial Clo-lip treatment and stained with anti-hCD235a and anti-hCD71 antibodies. Representative FACS plots are shown with the frequency of human CD235a^+^ cells in total RBC **(B)**. The frequencies of CD71^+^ immature hRBC in human CD235a^+^ hRBCs are shown with the representative FACS histograms **(C)**.

Long-term maintenance of transfused hRBCs in NOG-C3^ΔMG2-3^ mice allowed for examination of the differentiation of hRBCs from hHSCs. Our preliminary experiments suggested that Clo-lip, but not GdCl_3_, induced a few hRBCs (not greater than 1% in PB) in hHSC-NOG-C3^ΔMG2-3^ mice. Thus, we used NOG-C3^ΔMG2-3^/hGM-CSF-IL-3 (hGM3) Tg mice to enhance erythropoiesis and human myelopoiesis (15). The proportion of hRBCs in some hHSC-NOG-C3^ΔMG2-3^/hGM3 Tg mice reached approximately 8-10% in PB after three-time injections of Clo-lip. However, nearly half of the mice both in the NOG-C3^ΔMG2-3^/hGM3 Tg and hHSC-NOG/hGM3 Tg groups died during the experiments, resulting in a large variance and the absence of a statistical significance between these two groups (**Figure 7A**). The BM analysis showed an enhanced development of human erythrocytes in the NOG-C3^ΔMG2-3^/hGM3 Tg and hHSC-NOG/hGM3 Tg mice (**Figure 7B**). About 40% of them were CD71^+^ CD235a^+^ immature erythrocytes, indicating the erythropoiesis from hHSC in the BM (**Figure 7C**).

## Discussion

In this study, we investigated the molecular mechanisms underlying the rapid elimination of hRBCs from mouse circulation and identified mouse C3 as a critical mediator of hRBC depletion. We generated a C3-deficient NOG mouse strain, NOG-C3^ΔMG2-3^, in which clearance of transfused hRBCs was significantly slower than in NOG mice. Chemical depletion or suppression of mouse macrophages in NOG-C3^ΔMG2-3^ mice further extended the survival of transfused hRBCs; thus, repeated administration of less toxic GdCl_3_ and hRBCs produced stable hRBC-bearing NOG-C3^ΔMG2-3^ mice for more than 4 weeks.

The intolerance to hRBCs of NOG mice highlights the ability of innate immune cells to recognize xenogeneic cells and tissue. The innate immune system captures a wide variety of exogenous and “self-derived” antigens *via* multi-layered molecular systems involving Toll-like, lectin, and scavenger receptors. We hypothesized that the transfused hRBCs were exposed to soluble innate factors in the circulation, thereby contributing to their rapid clearance. The results showed deposition of mouse complement molecules on hRBCs, indicating that these molecules act as opsonins covering the surfaces of transfused hRBCs. A role of complement factors in hRBC elimination has previously been suggested (30). Chen et al. also recently reported that murine complement depletion by cobra venom factor (CVF) significantly prolonged the survival of infused hRBCs in NOD/SCID and NOD/SCID β2m-deficient mice treated with Clo-lip (25). In our NOG-C3^ΔMG2-3^ mice, genetic C3 depletion was sufficient to significantly extend the survival of hRBCs without Clo-lip treatment. CVF treatment may not completely eliminate complement molecules in mice.

Despite the prolonged survival of hRBCs in NOG-C3^ΔMG2-3^ and macrophage-depleted NOG mice, the hRBC lifespan in the mice was significantly shorter than the natural lifespan of hRBCs (up to 120 days). Notably, there was a synergistic effect between C3 deficiency and depletion of mouse macrophages. This was most evident during the initial 4 days after hRBC transfusion. NOG-C3^ΔMG2-3^ and Clo-lip-treated NOG mice showed a 50% reduction of hRBCs at 4 dpt, while there was a 15–20% reduction in Clo-lip-treated NOG-C3^ΔMG2-3^ mice; this synergy indicates that two major mechanisms, macrophage-dependent and C3-dependent, independently promote hRBC elimination in NOG mice. Considering the expression of C3 receptors in macrophages, these two mechanisms would be partially, but not totally, overlapped. In addition, a significant decay of hRBCs in Clo-lip-treated NOG-C3^ΔMG2-3^ mice in the early phase after Clo-lip treatment indicates that mechanisms independent of C3 or macrophages are likely involved to some extent. Because macrophages recover around 7 days after depletion by Clo-lip, hRBCs would be exposed to macrophage-dependent mechanisms and eliminated in the later phase.

The histological analysis of hRBC distribution in NOG mice showed engulfment of hRBCs by mouse CD68^+^ macrophages in the spleen and liver at 24 hours after hRBC transfusion (**Supplementary Figure 6**), indicating that transfused hRBCs are trapped in the reticuloendothelial system. The molecular mechanisms underlying the engulfment by macrophages remain to be clarified. Humoral molecules, including C3, may facilitate phagocytosis as opsonins. In addition, macrophages may have unique receptors that directly recognize surface antigens on hRBCs. Siglec-1 was previously reported to bind xenogeneic RBCs (39, 40) and this mechanism may be responsible for the elimination of hRBCs. Regarding C3-dependent mechanisms, because NOD mice lack a functional C5 gene due to the hemolytic complement (*Hc*) mutation (41, 42), the effects of C3 on hRBC clearance could be mediated by cellular mechanisms rather than complement cascade-dependent lysis, which requires the activation of C5–C9 molecules. C3 may induce phagocytic cells, including both macrophages and non-macrophages, to eliminate hRBCs. Alternatively, C3-dependent mechanisms may sequester hRBCs in some tissues independent of phagocytosis, although this remains to be clarified.

Treatment with chemical compounds to suppress mouse macrophages is required to maximize the utility of hRBC-infused NOG-C3^ΔMG2-3^ mouse models. In the present study, hRBCs were maintained in NOG-C3^ΔMG2-3^ mice for nearly one month by weekly injections of hRBCs and GdCl_3._ Since, current protocols for maintaining hRBCs in immunodeficient mice require daily injections of hRBCs, our model practically facilitates the production and handling of hRBC-engrafted mouse models. In addition, the time required for replenishment of hRBCs in NOG-C3^ΔMG2-3^ mice is significantly longer than that in conventional NOG mice, which facilitates recapitulation of the erythrocytic cycle of malaria parasites, in which infection and rupture of hRBCs occur repeatedly.

The comparison of three different mouse strains with hHSCs indicates that there are several requirements for establishing hRBC-engrafted models. Firstly, the enhanced difference of human erythropoiesis in NOG-C3^ΔMG2-3^/hGM3 Tg or NOG/hGM3Tg mice compared to NOG-C3^ΔMG2-3^ mice indicates that human GM-CSF and IL-3 play a dominant role in human erythropoiesis in the BM. Secondly, the relatively higher frequency of mature hRBCs in the PB in NOG-C3^ΔMG2-3^/hGM3 Tg than in NOG/hGM3Tg mice showed the importance of C3 deficiency. Considering the similar level of erythropoiesis in the BM in these two strains, however, C3 deficiency would facilitate accumulation of hRBCs in the periphery rather than promoting erythropoiesis. In addition to these two requirements, strong elimination of mouse macrophages is essential. It would be supportive that a paper by Song demonstrated that simultaneous transplantation of human liver and HSC achieved the development of hRBC from human HSC in MISTRG mice with the additive genetic deficiency in the fumarylacetoacetate hydrolase gene (*Fah* ^-/-^) (23). This model strongly enhances human hematopoiesis by human M-CSF, IL-3, GM-CSF, and thrombopoietin (9). In addition, after reconstitution of human liver, the dual chimera mice with human liver had decrease amounts of mouse C3 and mouse Kupffer cells. Hence, this model and ours have some similarities. Indeed, chimeric ratio of hRBC in the PB was close between these two models.

In conclusion, NOG-C3^ΔMG2-3^ mice were shown to be suitable for producing a long-term hRBC-bearing mouse model. Further studies are needed to identify the molecular mechanisms underlying macrophage-mediated hRBC elimination, for the generation of better mouse models in which hRBCs can survive for an extended period, ideally close to their natural life span, without using Clo-lip or GdCl_3_.

## Data Availability Statement

The raw data supporting the conclusions of this article will be made available by the authors, without undue reservation.

## Ethics Statement

The studies involving human participants were reviewed and approved by The research ethics committee of the CIEA. The participants provided their written informed consent to participate in this study.

## Author Contributions

TY, IO and TT designed the project, conducted experiments, and completed the manuscript. MM performed the pathological examinations. MG performed the embryo manipulation. IK and RI contributed to the critical reading of the manuscript. All authors contributed to the article and approved the submitted version,

## Funding

This work was supported in part by a Grant-in-Aid for Scientific Research (B) (18H02368 to TT) and Grant-in-Aid for Early-Career Scientists (20K15704 to TY). This project was commissioned by a Grant-in-Aid for Research on Hepatitis from the Japan Agency for Medical Research and Development.

## Conflict of Interest

The authors declare that the research was conducted in the absence of any commercial or financial relationships that could be construed as a potential conflict of interest.

## Publisher’s Note

All claims expressed in this article are solely those of the authors and do not necessarily represent those of their affiliated organizations, or those of the publisher, the editors and the reviewers. Any product that may be evaluated in this article, or claim that may be made by its manufacturer, is not guaranteed or endorsed by the publisher.
