# Supplementary material for: Generation of Novel Human Red Blood Cell-Bearing Humanized Mouse Models Based on C3-Deficient NOG Mice
Source: Front Immunol. 2021 Jul 27;12:671648. doi: 10.3389/fimmu.2021.671648 (PMC8353390; doi:10.3389/fimmu.2021.671648)
Supplement: Supplementary file 1 [file DataSheet_1.docx]

Supplementary Material


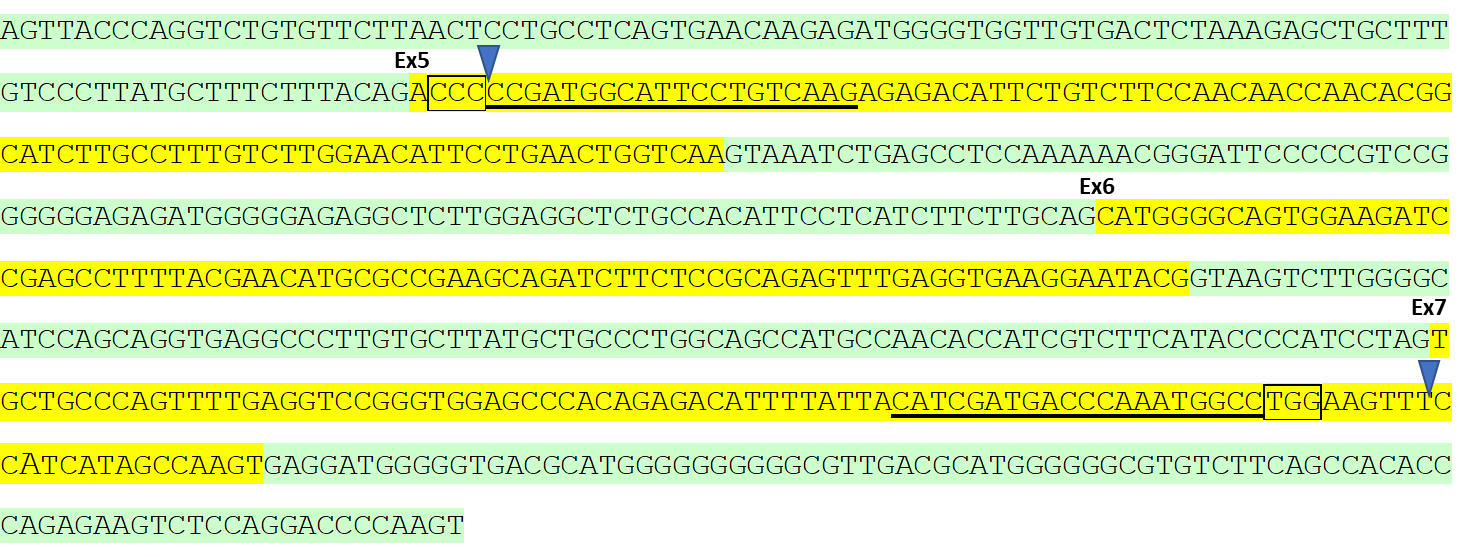


**Supplementary Figure 1.** **Genome Editing of the mouse C3 gene.**

The guide RNAs were underlined with the protospacer adjacent motif (PAM) sequences. Arrow heads indicate the cleavage sites.


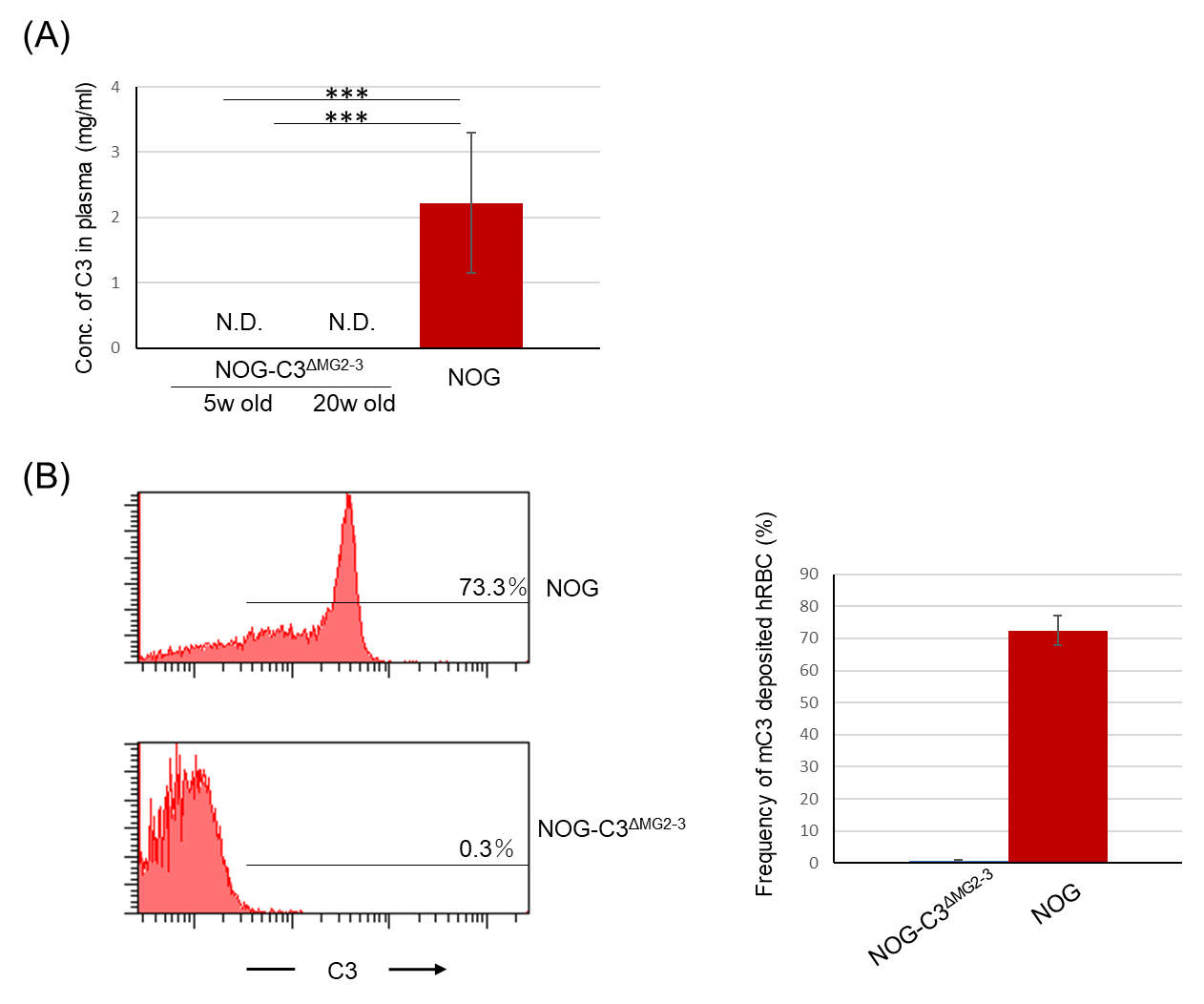


**Supplementary Figure 2**. **Generation of the C3-deficient NOG substrain NOG-C3^ΔMG2-3^.**

(A) Absence of mouse C3 in NOG-C3^ΔMG2-3^ mice. Plasma samples were collected from NOG-C3^ΔMG2-3^ mice (5 and 20 weeks old, n = 5 for each group) and their parental NOG mice (n = 5). Mouse C3 levels were measured using ELISA. Data are presented as the means ± SDs. Asterisks indicate the statistical significance determined using one-way ANOVA (***p < 0.001). (B) Failure of deposition of mouse C3 on hRBCs *in vitro*. The hRBCs were incubated with serum from NOG (n = 3) or NOG-C3^ΔMG2-3^ (n = 6) mice *in vitro* and stained with anti-mouse C3 antibody. A representative histogram is shown.

**
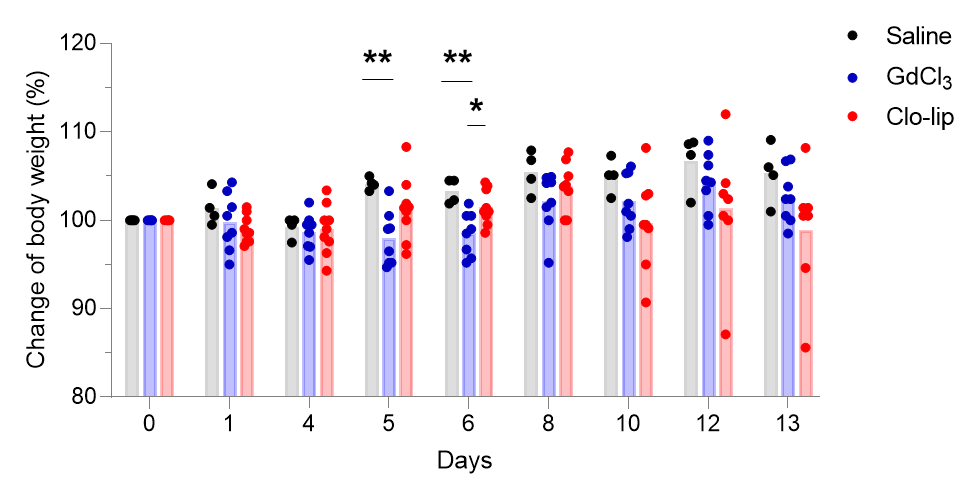
**

**Supplementary Figure 3. Effects of Clo-lip and GdCl_3_ on mouse health and hRBCs in NOG mice.**

Dots represent the weights of individual mice, and bar graphs represent the means. Asterisks indicate statistically significant differences as determined by Mixed-effects analysis (**p < 0.01 and *p < 0.05).

**
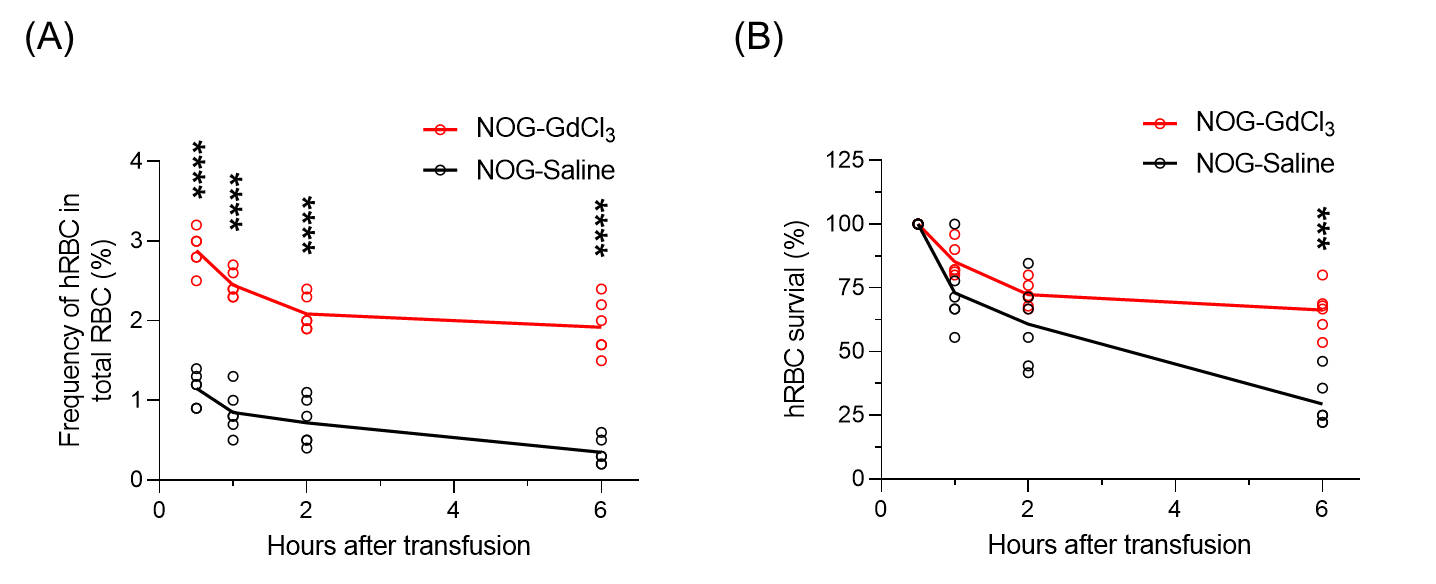
**

**Supplementary Figure 4. Prolonged survival of hRBCs in NOG mice treated with GdCl_3_.**

NOG mice were treated with GdCl_3_ four times at 3–4-day intervals. Mice were then transfused with hRBCs 1 day after the last treatment. Frequencies (A) and survival rates (B) of transfused hRBCs in NOG mice. Each group consisted of six mice. Asterisks indicate statistically significant differences between GdCl_3_-injected mice and saline-injected mice, which were determined by two-way repeated-measures ANOVA (****p < 0.0001 and ***p < 0.001).

**
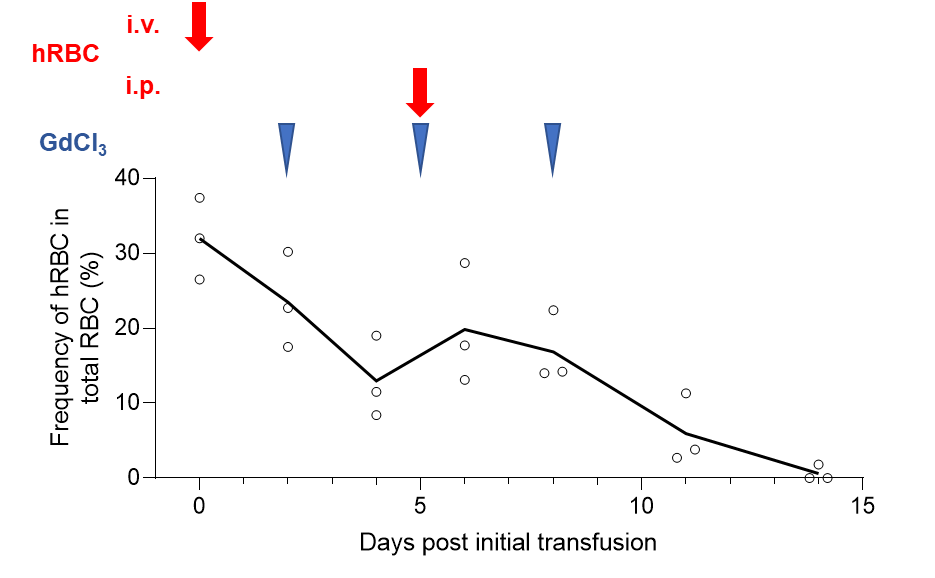
**

**Supplementary Figure 5. Repeated injections of GdCl_3_ to hRBC-bearing NOG mice.**

NOG mice were treated with Clo-lip once, and subsequently with GdCl_3_ three times at 3–4-day intervals. Mice were transfused with i.v. (5.0 × 10^9^) injections of hRBCs on the next day of the pretreatment cycle. GdCl_3_ was administered every 3 days to suppress murine macrophages. hRBCs were i.p.- injected on day 5.


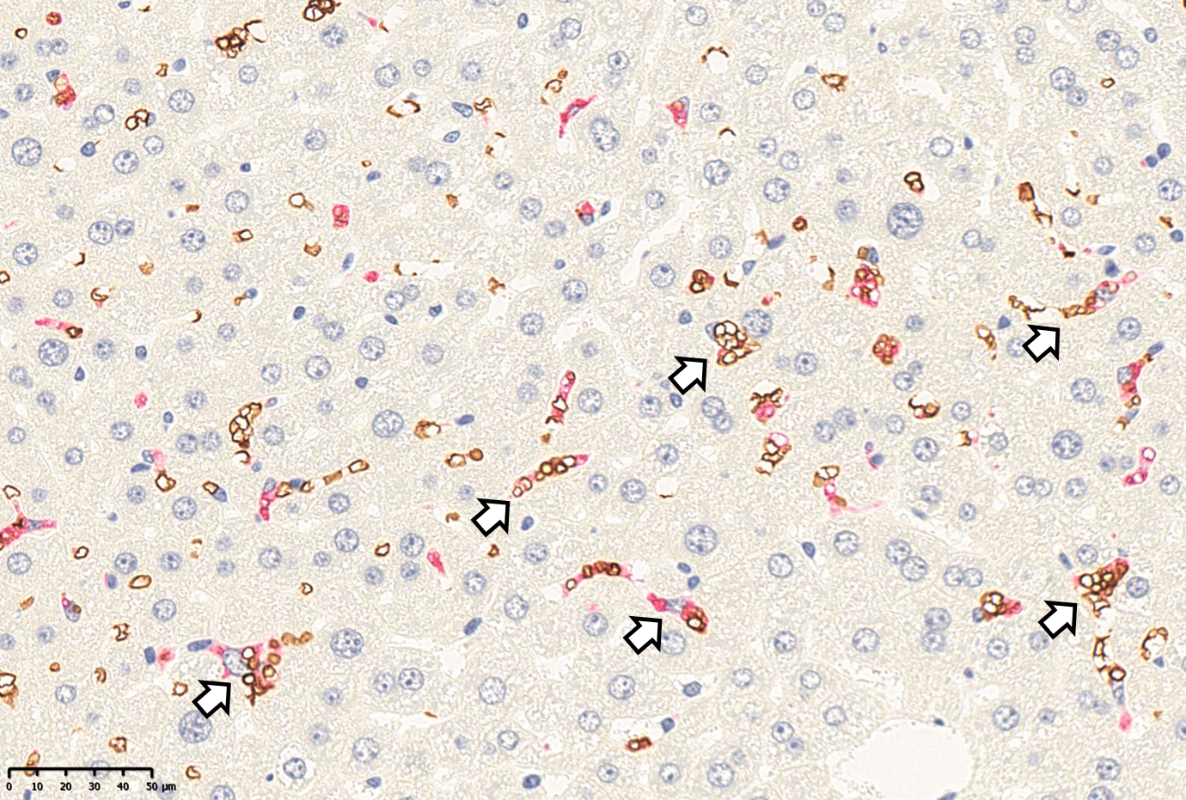
(A)


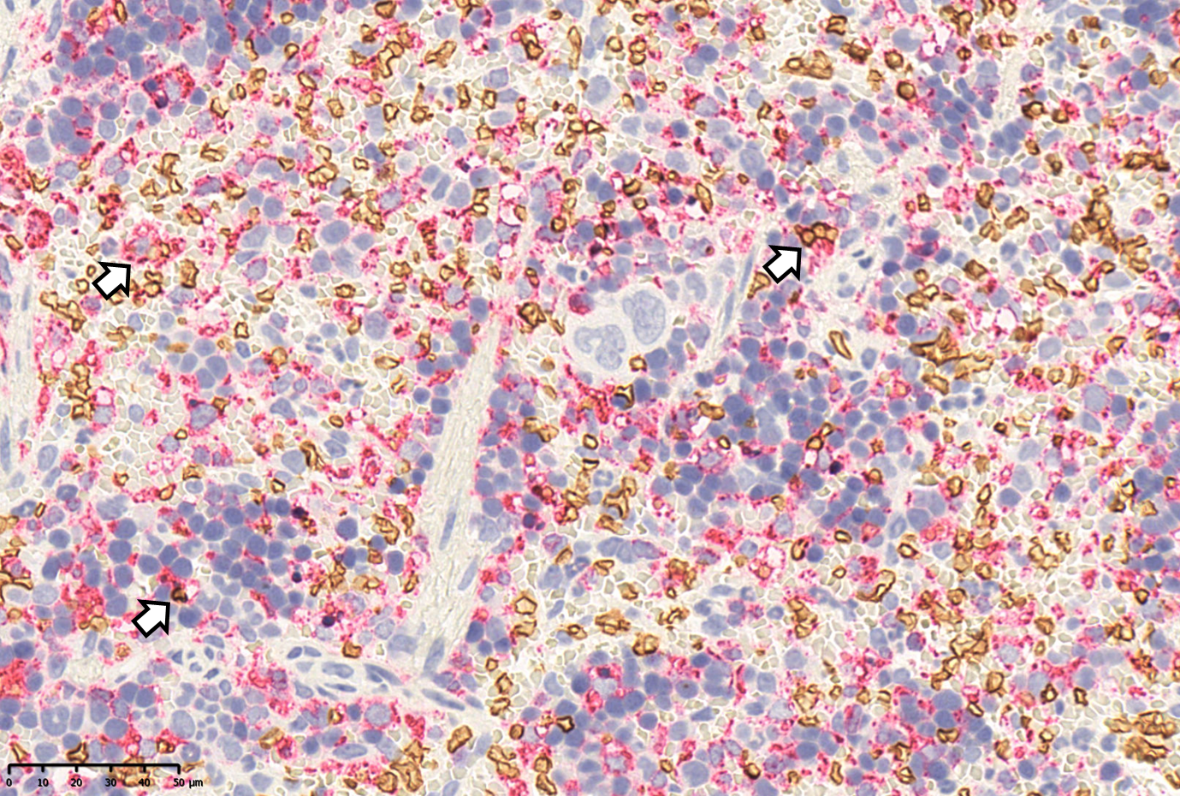
(B)

**Supplementary Figure 6. Engulfment of human RBC by mouse liver and splenic macrophages.**

Liver (A) and spleen (B) were collected from NOG mice 24 hours after transfusion with hRBCs. The sections were stained with anti-mouse CD68 (red) for mouse macrophages and anti-GlyA (brown) for human RBCs. Anti-mouse CD68 was developed by Bond^TM^ Polymer Refine Red Detection kit using Fast Red and alkaline phosphatase (AP). Anti-GlyA was developed by Bond^TM^ Polymer Refine Detection kit using diaminobenzidine (DAB) and horse radish peroxidase (HRP). White arrows indicate the engulfment of hRBCs by mouse macrophages.
